# A Merited Compliment

**Published:** 1886-04

**Authors:** 


### A MERITED COMPLIMEMT.

It is the custom with some medical colleges to annually select some one, not a graduate of that school, distinguished for services in the cause of medicine, and to confer upon him the Honorary Degree of Doctor of Medicine, in recognition of those services. This honor is always unsolicited, and has never been awarded an applicant.

Recently the Louisville Medical College selected for the purpose, our own enlightened sanitarian and physician, Dr. R. M. Swearengen, the well known State Health Officer of Texas, and conferred upon him that degree.

In this selection the Louisville Medical College is to be congratulated. The faculty could not have made a more judicious choice; nor could they have more worthily bestowed this high honor. It has been placed where it will be worn with distinguished credit to the faculty, the college, and to the State of Kentucky; and Texas joins hands with Kentucky in doing honor to the gallant Mississippian, her adopted "son, in whom she is well plased." Long may he live to serve the profession he so adorns, and to shed lustre upon his foster mother!

